# Dyakonov–Voigt surface waves

**DOI:** 10.1098/rspa.2019.0317

**Published:** 2019-08-28

**Authors:** Tom G. Mackay, Chenzhang Zhou, Akhlesh Lakhtakia

**Affiliations:** 1 School of Mathematics and Maxwell Institute for Mathematical Sciences, University of Edinburgh, Edinburgh EH9 3FD, UK; 2 NanoMM—Nanoengineered Metamaterials Group, Department of Engineering Science and Mechanics, Pennsylvania State University, University Park, PA 16802-6812, USA

**Keywords:** Dyakonov surface waves, Voigt waves, singular optics

## Abstract

Electromagnetic surface waves guided by the planar interface of an isotropic dielectric medium and a uniaxial dielectric medium, both non-dissipative, were considered, the optic axis of the uniaxial medium lying in the interface plane. Whereas this interface is known to support the propagation of Dyakonov surface waves when certain constraints are satisfied by the constitutive parameters of the two partnering mediums, we identified a different set of constraints that allow the propagation of surface waves of a new type. The fields of the new surface waves, named Dyakonov–Voigt (DV) surface waves, decay as the product of a linear and an exponential function of the distance from the interface in the anisotropic medium, whereas the fields of the Dyakonov surface waves decay only exponentially in the anisotropic medium. In contrast to Dyakonov surface waves, the wavenumber of a DV surface wave can be found analytically. Also, unlike Dyakonov surface waves, DV surface waves propagate only in one direction in each quadrant of the interface plane.

## 1. Introduction

The planar interface of two dissimilar mediums, labelled A and B, supports the propagation of electromagnetic surface waves. A variety of different types of such surface waves can be guided by planar interfaces, depending upon the nature of the two partnering mediums [1,2]. In this paper, the focus is on the planar interface of two dielectric mediums, medium A being anisotropic and medium B being isotropic. For certain constitutive parameter regimes, this interface supports the propagation of Dyakonov surface waves, as is well established both theoretically [3,4] and experimentally [5]. Unlike other types of surface waves, such as the widely studied surface–plasmon–polariton waves [2,6,7], Dyakonov surface waves propagate without decay when both partnering mediums are non-dissipative [8,9]. Accordingly, these surface waves represent attractive propositions for applications involving long-range optical communications. Generally, these surface waves can propagate only for a small range of angular directions parallel to the interface plane, typically only a few degrees [8,10], albeit much larger angular existence domains for Dyakonov surface waves can be achieved using dissipative partnering mediums [11,12].

A convenient formalism for analysing electromagnetic surface waves involves a 4 × 4 characteristic matrix denoted by $\left[\underline{\underline{M}}\right]$ [2]. The dispersion relation for surface waves is obtained by equating the determinant of $\left[\underline{\underline{M}}\right]$ to zero. The characteristic matrix comprises four column vectors. Two of the column 4-vectors are eigenvectors of a 4 × 4 propagation matrix $\left[{\underline{\underline{P}}}_{A}\right]$ for medium A, and the remaining two are eigenvectors of a 4 × 4 propagation matrix $\left[{\underline{\underline{P}}}_{B}\right]$ for medium B [13]. These eigenvectors are chosen to ensure that the fields of the surface wave decay with distance from the planar interface. In the well-established case of Dyakonov surface-wave propagation [2–4], both column 4-vectors provided by $\left[{\underline{\underline{P}}}_{A}\right]$ are linearly independent of each other and both column 4-vectors provided by $\left[{\underline{\underline{P}}}_{B}\right]$ are linearly independent of each other.

In this paper, we consider surface-wave propagation when the two column 4-vectors provided by $\left[{\underline{\underline{P}}}_{A}\right]$ are *not* linearly independent of each other. As is shown in the following sections, the corresponding surface wave—which we call a Dyakonov–Voigt (DV) surface wave—is fundamentally different from a Dyakonov surface wave insofar as the amplitude of a DV surface wave decays in a combined exponential–linear manner with increasing distance from the interface in medium A. Also, a DV surface wave propagates in only one direction in each quadrant of the interface plane, unlike a Dyakonov surface wave that propagates for a range of directions in each quadrant of the interface plane [8,9].

We decided to name these new surface waves after both Dyakonov and Voigt, because the surface-wave fields in the partnering medium A are closely related to a singular form of planewave propagation known as Voigt-wave propagation [14]. A Voigt wave propagates in an unbounded anisotropic medium when the corresponding propagation matrix cannot be diagonalized [15,16]. Unlike the conventional plane waves that propagate in unbounded anisotropic mediums [17,18], the amplitude of a Voigt wave is the product of an exponential function of the propagation distance and a linear function of the propagation distance. Early experimental and theoretical studies on Voigt waves were based on pleochroic crystals such as iolite [14,15,19]. More recently, the greater scope for realizing Voigt-wave propagation in more complex mediums [20,21], including bianisotropic [22] and non-homogeneous mediums [23], has been reported. In particular, through judicious design, engineered materials can be used to allow control over the directions for Voigt-wave propagation [24–28]. An essential requirement for Voigt-wave propagation is that the host anisotropic medium is either dissipative [16,19,29] or active [30]; but in the case of DV surface-wave propagation, as described in greater detail the following sections, the two partnering mediums are both non-dissipative and inactive.

In the following sections, the theory of DV surface-wave propagation is developed by solving a canonical boundary-value problem to formulate $\left[\underline{\underline{M}}\right]$. In order to provide context, a brief description is also provided of Dyakonov surface-wave propagation [2,4]. Constraints on the constitutive-parameter regimes that allow DV surface-wave propagation are established and explicit analytic solutions of the dispersion relation for DV surface waves are presented. These theoretical results are illustrated by means of representative numerical results. Some closing remarks are offered in the final section.

As regards notation, 3 × 3 dyadics are denoted by double underlining and 3-vectors are denoted by single underlining; 4 × 4 matrixes are denoted by double underlining and square parenthesis and column 4-vectors are denoted by single underlining and square parenthesis. The triad of Cartesian basis vectors is $\left\{{\hat{\underline{u}}}_{x},{\hat{\underline{u}}}_{y},{\hat{\underline{u}}}_{z}\right\}$. The identity 3 × 3 dyadic is $\underline{\underline{I}}={\hat{\underline{u}}}_{x}{\hat{\underline{u}}}_{x}+{\hat{\underline{u}}}_{y}{\hat{\underline{u}}}_{y}+{\hat{\underline{u}}}_{z}{\hat{\underline{u}}}_{z}$. The permittivity and permeability of free space are *ε*_0_ = 8.854 × 10^−12^ F m^−1^ and *μ*_0_ = 4*π* × 10^−7^ H m^−1^, respectively. The free-space wavenumber is written as $k_{0}=\omega \sqrt{\varepsilon_{0}\mu_{0}}$, with *ω* being the angular frequency; the free-space wavelength and impedance are *λ*_0_ = 2*π*/*k*_0_ and $\eta_{0}=\sqrt{\mu_{0}/\varepsilon_{0}}$, respectively. Also, $i=\sqrt{-1}$.

## 2. Canonical boundary-value problem

We specialize a general formalism [2] to develop the canonical boundary-value problem for surface-wave propagation guided by the planar interface of a uniaxial dielectric medium A and an isotropic dielectric medium B. Filling the half-space *z* > 0, medium A is characterized by the relative permittivity dyadic [17,18]

**Figure 1.**
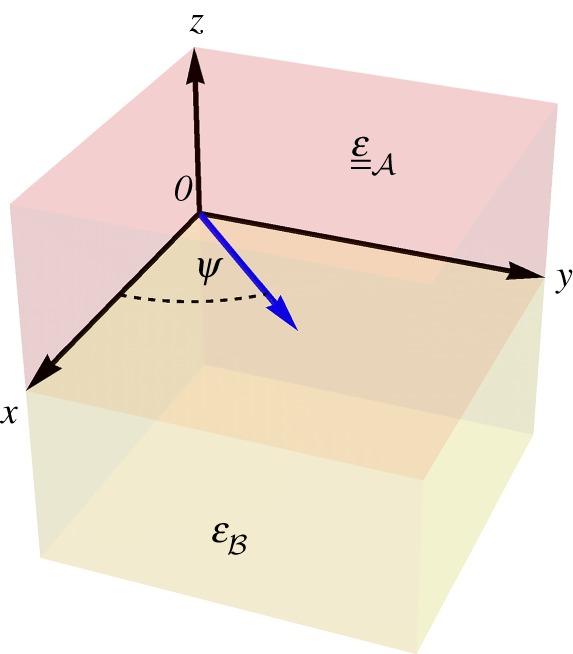
A schematic of the canonical boundary-value problem. The optic axis of medium A is parallel to the *x*-axis. Surface waves propagate parallel to the interface plane *z* = 0, at the angle *ψ* relative to the *x*-axis. (Online version in colour.)

2.1 $${\underline{\underline{\varepsilon }}}_{A}=\varepsilon_{A}^{s}\underline{\underline{I}}+\left(\varepsilon_{A}^{t}-\varepsilon_{A}^{s}\right){\hat{\underline{u}}}_{x}\;{\hat{\underline{u}}}_{x},$$

with the unit vector ${\hat{\underline{u}}}_{x}$ pointing in the direction of the optic axis of medium A. The dyadic ${\underline{\underline{\varepsilon }}}_{A}$ has two eigenvalues: $\varepsilon_{A}^{s}$ of algebraic multiplicity [31] equal to 2 and $\varepsilon_{A}^{t}$ of algebraic multiplicity equal to 1. When medium A is considered to fill all space, the propagation of *ordinary* plane waves is governed by $\varepsilon_{A}^{s}$, while the propagation of *extraordinary* plane waves is governed by both $\varepsilon_{A}^{s}$ and $\varepsilon_{A}^{t}$ [32]. The isotropic medium B fills the half-space *z* < 0 and is characterized by the relative permittivity dyadic ${\underline{\underline{\varepsilon }}}_{B}=\varepsilon_{B}\underline{\underline{I}}$. Both mediums are non-magnetic and non-magnetoelectric [18,22,33]. A schematic of the canonical boundary-value problem is presented in figure 1.

The electromagnetic field phasors of a surface wave can be written for all *z* as [2]

2.2 $$\begin{array}{rl} \underline{E}(\underline{r}) & =\underline{e}(z)\;\exp\left[iq\left(x\cos\psi +y\sin\psi \right)\right]\\ \;\mathrm{and}\;\underline{H}(\underline{r}) & =\underline{h}(z)\;\exp\left[iq\left(x\cos\psi +y\sin\psi \right)\right], \end{array}\}$$

where *q* is the surface wavenumber. The angle *ψ*∈[0, 2*π*) specifies the direction of propagation in the *xy*-plane, relative to the *x*-axis. The auxiliary phasors may be written as

2.3 $$\begin{array}{rl} & \underline{e}(z)=e_{x}(z){\hat{\underline{u}}}_{x}+e_{y}(z){\hat{\underline{u}}}_{y}+e_{z}(z){\hat{\underline{u}}}_{z}\\ \;\mathrm{and}\; & \underline{h}(z)=h_{x}(z){\hat{\underline{u}}}_{x}+h_{y}(z){\hat{\underline{u}}}_{y}+h_{z}(z){\hat{\underline{u}}}_{z}. \end{array}\}$$

The spatial profiles of the field phasors (2.2) are governed by the source-free, frequency-domain Maxwell curl postulates [17]

2.4 $$\begin{array}{rl} & \nabla \times \underline{H}(\underline{r},\omega )+i\omega \varepsilon_{0}{\underline{\underline{\varepsilon }}}_{A}\cdot \underline{E}(\underline{r},\omega )=\underline{0}\\ \;\mathrm{and}\; & \nabla \times \underline{E}(\underline{r},\omega )-i\omega \mu_{0}\underline{H}(\underline{r},\omega )=\underline{0} \end{array}\},\;z>0$$

and

2.5 $$\begin{array}{rl} & \nabla \times \underline{H}(\underline{r},\omega )+i\omega \varepsilon_{0}\varepsilon_{B}\underline{E}(\underline{r},\omega )=\underline{0}\\ \;\mathrm{and}\; & \nabla \times \underline{E}(\underline{r},\omega )-i\omega \mu_{0}\underline{H}(\underline{r},\omega )=\underline{0} \end{array}\},\;z<0.$$

When combined with the phasor representations (2.2), the Maxwell curl postulates (2.4) and (2.5), respectively, yield the 4 × 4 matrix ordinary differential equations

2.6 $$\frac{d}{dz}\left[\underline{f}(z)\right]=i[{\underline{\underline{P}}}_{A}]\cdot \left[\underline{f}(z)\right],\;z>0,$$

and

2.7 $$\frac{d}{dz}\left[\underline{f}(z)\right]=i[{\underline{\underline{P}}}_{B}]\cdot \left[\underline{f}(z)\right],\;z<0,$$

where the form of the 4 × 4 propagation matrix $\left[{\underline{\underline{P}}}_{\ell }\right]$, ℓ∈{A,B}, depends upon the form of ${\underline{\underline{\varepsilon }}}_{\ell }$. The column 4-vector

2.8 $$\left[\underline{f}(z)\right]=\left[\begin{array}{c} e_{x}(z)\\ e_{y}(z)\\ h_{x}(z)\\ h_{y}(z) \end{array}\right],$$

contains the *x*-directed and *y*-directed components of the auxiliary phasors. These components are algebraically connected to the *z*-directed components of the auxiliary phasors [18].

### (a) Half-space *z* > 0

Relevant to the half-space *z* > 0,

2.9 $$\begin{array}{r} \left[{\underline{\underline{P}}}_{A}\right]=\left[\begin{array}{cccc} 0 & 0 & \frac{q^{2}\cos\psi \sin\psi }{\omega \varepsilon_{0}\varepsilon_{A}^{s}} & \frac{k_{0}^{2}\varepsilon_{A}^{s}-q^{2}\cos^{2}\psi }{\omega \varepsilon_{0}\varepsilon_{A}^{s}}\\ 0 & 0 & \frac{-k_{0}^{2}\varepsilon_{A}^{s}+q^{2}\sin^{2}\psi }{\omega \varepsilon_{0}\varepsilon_{A}^{s}} & -\frac{q^{2}\cos\psi \sin\psi }{\omega \varepsilon_{0}\varepsilon_{A}^{s}}\\ -\frac{q^{2}\cos\psi \sin\psi }{\omega \mu_{0}} & \frac{-k_{0}^{2}\varepsilon_{A}^{s}+q^{2}\cos^{2}\psi }{\omega \mu_{0}} & 0 & 0\\ \frac{k_{0}^{2}\varepsilon_{A}^{t}-q^{2}\sin^{2}\psi }{\omega \mu_{0}} & \frac{q^{2}\cos\psi \sin\psi }{\omega \mu_{0}} & 0 & 0 \end{array}\right], \end{array}$$

while the *z*-directed components of the auxiliary phasors are given by

2.10 $$\begin{array}{rl} & e_{z}(z)=\frac{q\left[h_{x}(z)\sin\psi -h_{y}(z)\cos\psi \right]}{\omega \varepsilon_{0}\varepsilon_{A}^{s}}\\ \;\mathrm{and}\; & h_{z}(z)=\frac{q\left[e_{y}(z)\cos\psi -e_{x}(z)\sin\psi \right]}{\omega \mu_{0}} \end{array}\},\;z>0.$$

#### (i) Non-singular case.

In the non-singular case, $\left[{\underline{\underline{P}}}_{A}\right]$ has four eigenvalues, each with algebraic multiplicity 1 and geometric multiplicity 1. We name these $\pm \alpha_{A1}$ and $\pm \alpha_{A2}$, where

2.11 $$\begin{array}{rl} & \alpha_{A1}=i\sqrt{q^{2}-k_{0}^{2}\varepsilon_{A}^{s}}\\ \;\mathrm{and}\; & \alpha_{A2}=i\sqrt{\frac{q^{2}\left[\left(\varepsilon_{A}^{s}+\varepsilon_{A}^{t}\right)-\left(\varepsilon_{A}^{s}-\varepsilon_{A}^{t}\right)\cos2\psi \right]-2k_{0}^{2}\varepsilon_{A}^{s}\varepsilon_{A}^{t}}{2\varepsilon_{A}^{s}}}. \end{array}\}$$

In order to conform to a surface-wave representation, the signs of the square root terms in equations (2.11) are selected such that $\text{Im}\{\alpha_{A1}\}>0$ and $\text{Im}\{\alpha_{A2}\}>0$. A pair of eigenvectors of the 4 × 4 matrix $\left[{\underline{\underline{P}}}_{A}\right]$ corresponding to the eigenvalues $\alpha_{A1}$ and $\alpha_{A2}$ are

2.12 $$\begin{array}{rl} & {\underline{v}}_{A1}=\left[\begin{array}{c} 0\\ \frac{k_{0}\alpha_{A1}}{q^{2}\sin\psi \cos\psi }\\ \frac{\cot2\psi }{\eta_{0}}+\frac{\csc2\psi }{\eta_{0}}\left(1-\frac{2k_{0}^{2}\varepsilon_{A}^{s}}{q^{2}}\right)\\ \eta_{0}^{-1} \end{array}\right]\\ \;\mathrm{and}\; & {\underline{v}}_{A2}=\left[\begin{array}{c} 1-\frac{q^{2}\left(\cos2\psi +1\right)}{2k_{0}^{2}\varepsilon_{A}^{s}}\\ -\frac{q^{2}\cos\psi \sin\psi }{k_{0}^{2}\varepsilon_{A}^{s}}\\ 0\\ \frac{\alpha_{A2}}{\omega \mu_{0}} \end{array}\right], \end{array}\}$$

respectively. Thus, the general solution to equation (2.6) for fields that decay as *z* →  + ∞ is given as

2.13 $$\left[\underline{f}(z)\right]=C_{A1}{\underline{v}}_{A1}\exp\left(i\alpha_{A1}z\right)+C_{A2}{\underline{v}}_{A2}\exp\left(i\alpha_{A2}z\right),\;z>0,$$

wherein the constants $C_{A1}$ and $C_{A2}$ are determined by the boundary conditions at *z* = 0.

#### (ii) Singular case.

The singular case arises when

2.14 $$q=\sigma \frac{k_{0}\sqrt{\varepsilon_{A}^{s}}}{\cos\psi },$$

where the sign parameter *σ* =  + 1 for *ψ*∈(0, *π*/2) and *σ* =  − 1 for *ψ*∈(*π*/2, *π*). Then $\left[{\underline{\underline{P}}}_{A}\right]$ has only two eigenvalues, each with algebraic multiplicity 2 and geometric multiplicity 1. We name these $\pm \alpha_{A}$, where

2.15 $$\alpha_{A}=i\sigma k_{0}\sqrt{\varepsilon_{A}^{s}}\tan\psi ,$$

with the square root selected to have a positive real part in order to achieve $\text{Im}\{\alpha_{A}\}>0$, which is essential for surface-wave propagation [2]. Since $\text{Im}\{\alpha_{A}\}\leq 0$ for *ψ*∈{0, *π*}, DV surface-wave propagation is not possible for *ψ* = 0 and *π*.

An eigenvector of matrix $\left[{\underline{\underline{P}}}_{A}\right]$ corresponding to the eigenvalue $\alpha_{A}$ is

2.16 $${\underline{v}}_{A}=\left[\begin{array}{c} 0\\ \frac{i\sigma }{\sqrt{\varepsilon_{A}^{s}}}\\ 0\\ \eta_{0}^{-1} \end{array}\right];$$

and a corresponding generalized eigenvector that satisfies [34]

2.17 $$\left(\left[{\underline{\underline{P}}}_{A}\right]-\alpha_{A}\underline{\underline{I}}\right)\cdot {\underline{w}}_{A}={\underline{v}}_{A}$$

is

2.18 $${\underline{w}}_{A}=\frac{1}{k_{0}}\left[\begin{array}{c} \frac{2}{\varepsilon_{A}^{t}-\varepsilon_{A}^{s}}\\ \frac{\tan\psi }{\varepsilon_{A}^{s}}\left(\cot^{2}\psi -2\frac{\varepsilon_{A}^{s}-\varepsilon_{A}^{t}\cot^{2}\psi }{\varepsilon_{A}^{s}-\varepsilon_{A}^{t}}\right)\\ \frac{2i\sigma \sqrt{\varepsilon_{A}^{s}}}{\eta_{0}\left(\varepsilon_{A}^{t}-\varepsilon_{A}^{s}\right)}\\ 0 \end{array}\right].$$

Thus, the general solution of equation (2.6) for fields that decay as *z* →  + ∞ is given as

2.19 $$\left[\underline{f}(z)\right]=\left[C_{A1}{\underline{v}}_{A}+C_{A2}\left(iz\;{\underline{v}}_{A}+{\underline{w}}_{A}\right)\right]\exp\left(i\alpha_{A}z\right),\;z>0,$$

wherein the constants $C_{A1}$ and $C_{A2}$ are determined by the boundary conditions at *z* = 0.

### (b) Half-space *z* < 0

The 4 × 4 matrix $\left[{\underline{\underline{P}}}_{B}\right]$ is given as [2,17]

2.20 $$\begin{array}{r} \left[{\underline{\underline{P}}}_{B}\right]=\left[\begin{array}{cccc} 0 & 0 & \frac{q^{2}\cos\psi \sin\psi }{\omega \varepsilon_{0}\varepsilon_{B}} & \frac{k_{0}^{2}\varepsilon_{B}-q^{2}\cos^{2}\psi }{\omega \varepsilon_{0}\varepsilon_{B}}\\ 0 & 0 & \frac{-k_{0}^{2}\varepsilon_{B}+q^{2}\sin^{2}\psi }{\omega \varepsilon_{0}\varepsilon_{B}} & -\frac{q^{2}\cos\psi \sin\psi }{\omega \varepsilon_{0}\varepsilon_{B}}\\ -\frac{q^{2}\cos\psi \sin\psi }{\omega \mu_{0}} & \frac{-k_{0}^{2}\varepsilon_{B}+q^{2}\cos^{2}\psi }{\omega \mu_{0}} & 0 & 0\\ \frac{k_{0}^{2}\varepsilon_{B}-q^{2}\sin^{2}\psi }{\omega \mu_{0}} & \frac{q^{2}\cos\psi \sin\psi }{\omega \mu_{0}} & 0 & 0 \end{array}\right], \end{array}$$

while the *z*-directed components of the auxiliary phasors are given by

2.21 $$\begin{array}{rl} & e_{z}(z)=\frac{q\left[h_{x}(z)\sin\psi -h_{y}(z)\cos\psi \right]}{\omega \varepsilon_{0}\varepsilon_{B}}\\ \;\mathrm{and}\; & h_{z}(z)=\frac{q\left[e_{y}(z)\cos\psi -e_{x}(z)\sin\psi \right]}{\omega \mu_{0}} \end{array}\},\;z<0\;.$$

The 4 × 4 matrix $\left[{\underline{\underline{P}}}_{B}\right]$ has two eigenvalues, each with algebraic multiplicity 2 and geometric multiplicity 2. We name these $\pm \alpha_{B}$, where

2.22 $$\alpha_{B}=-i\sqrt{q^{2}-k_{0}^{2}\varepsilon_{B}}.$$

For surface-wave propagation, the sign of the square root in equation (2.22) is selected such that $\text{Im}\{\alpha_{B}\}<0$. A pair of independent eigenvectors of the 4 × 4 matrix $\left[{\underline{\underline{P}}}_{B}\right]$ corresponding to the eigenvalue $\alpha_{B}$ are

2.23 $$\begin{array}{rl} {\underline{v}}_{B1} & =\left[\begin{array}{c} 1-\frac{q^{2}\cos^{2}\psi }{k_{0}^{2}\varepsilon_{B}}\\ -\frac{q^{2}\cos\psi \sin\psi }{k_{0}^{2}\varepsilon_{B}}\\ 0\\ \frac{\alpha_{B}}{\omega \mu_{0}} \end{array}\right]\\ \;\mathrm{and}\;{\underline{v}}_{B2} & =\left[\begin{array}{c} \frac{q^{2}\cos\psi \sin\psi }{k_{0}^{2}\varepsilon_{B}}\\ -1+\frac{q^{2}\sin^{2}\psi }{k_{0}^{2}\varepsilon_{B}}\\ \frac{\alpha_{B}}{\omega \mu_{0}}\\ 0 \end{array}\right]. \end{array}\}$$

Thus, the general solution of equation (2.7) for fields that decay as *z* →  − ∞ is given as

2.24 $$\left[\underline{f}(z)\right]=\left(C_{B1}{\underline{v}}_{B1}+C_{B2}{\underline{v}}_{B2}\right)\exp\left(i\alpha_{B}z\right),\;z<0,$$

wherein the constants $C_{B1}$ and $C_{B2}$ are determined by the boundary conditions at *z* = 0.

### (c) Canonical boundary-value problem

#### (i) Dyakonov surface waves.

The continuity of tangential components of the electric and magnetic field phasors across the interface *z* = 0 imposes four conditions that are represented compactly as

2.25 $$\left[\underline{f}(0^{+})\right]=\left[\underline{f}(0^{-})\right].$$

Substitution of equations (2.13) and (2.24) in the foregoing equation leads to

2.26 $$\left[\underline{\underline{M}}\right]\cdot \left[\begin{array}{c} C_{A1}\\ C_{A2}\\ C_{B1}\\ C_{B2} \end{array}\right]=\left[\begin{array}{c} 0\\ 0\\ 0\\ 0 \end{array}\right],$$

wherein the 4 × 4 characteristic matrix $\left[\underline{\underline{M}}\right]$ must be singular for surface-wave propagation [2]. The dispersion equation $|[\underline{\underline{M}}]|=0$ is equivalent to the equation

2.27 $$k_{0}^{2}\varepsilon_{A}^{s}\left(\varepsilon_{A}^{s}\alpha_{B}-\varepsilon_{B}\alpha_{A1}\right)\left(\alpha_{B}-\alpha_{A2}\right)\tan^{2}\psi =\alpha_{A1}\left(\alpha_{B}-\alpha_{A1}\right)\left(\varepsilon_{A}^{s}\alpha_{B}\alpha_{A2}-\varepsilon_{B}\alpha_{A1}^{2}\right),$$

which can be solved numerically for *q*. The symmetry of equation (2.27) is such that if a Dyakonov surface wave exists for angle *ψ* = *ψ*^⋆^, then Dyakonov surface-wave propagation is also possible for *ψ* =  − *ψ*^⋆^ and *ψ* = *π* ± *ψ*^⋆^.

#### (ii) Dyakonov–Voigt surface waves.

The continuity of tangential components of the electric and magnetic field phasors across the interface *z* = 0 gives rise to equation (2.25). Substitution of equations (2.19) and (2.24) in equation (2.25) can be represented compactly as

2.28 $$[\underline{\underline{N}}]\cdot \left[\begin{array}{c} C_{A1}\\ C_{A2}\\ C_{B1}\\ C_{B2} \end{array}\right]=\left[\begin{array}{c} 0\\ 0\\ 0\\ 0 \end{array}\right],$$

wherein the 4 × 4 characteristic matrix $\left[\underline{\underline{N}}\right]$ must be singular for surface-wave propagation. The dispersion equation $|[\underline{\underline{N}}]|=0$ simplifies to

2.29 $$\begin{array}{r} \left[2\varepsilon_{A}^{s}\left(\varepsilon_{B}+\varepsilon_{A}^{s}\right)+\left(\varepsilon_{A}^{s}-\varepsilon_{B}\right)\left(\varepsilon_{A}^{s}+\varepsilon_{A}^{t}\right)\cot^{2}\psi \right]+2\sqrt{\varepsilon_{A}^{s}}\left(\varepsilon_{A}^{s}+\varepsilon_{B}\right)\sqrt{\varepsilon_{A}^{s}+\left(\varepsilon_{A}^{s}-\varepsilon_{B}\right)\cot^{2}\psi }=0. \end{array}$$

Notice that equation (2.29) cannot be satisfied for $\varepsilon_{A}^{s}=\varepsilon_{B}$, except in the pathological case $\varepsilon_{A}^{s}=\varepsilon_{B}=0$ which we disregard as unphysical. The symmetries of equation (2.29) mirror those of equation (2.27); that is, if a DV surface-wave solution exists for angle *ψ* = *ψ*^⋆^, then DV surface-wave solutions also exist for *ψ* =  − *ψ*^⋆^ and *ψ* = *π* ± *ψ*^⋆^.

### (d) Constraints on Dyakonov–Voigt surface-wave propagation

In keeping with the context in which Dyakonov surface waves are usually considered [3–5,8], let us concentrate on the crystal-optics regime for wherein all relative-permittivity parameters are real and positive; thus,

2.30 $$\begin{array}{r} \varepsilon_{A}^{s}>0\\ \varepsilon_{A}^{t}>0\\ \;\mathrm{and}\;\varepsilon_{B}>0. \end{array}\}$$

Since the possibility of $\varepsilon_{A}^{s}=\varepsilon_{B}$ has already been discounted, the only remaining possibilities are $\varepsilon_{A}^{s}<\varepsilon_{B}$ and $\varepsilon_{A}^{s}>\varepsilon_{B}$. However, by inspection, the dispersion equation (2.29) for DV surface waves cannot be satisfied for $\varepsilon_{A}^{s}>\varepsilon_{B}$. Therefore, the only remaining possibility is $\varepsilon_{A}^{s}<\varepsilon_{B}$.

The term enclosed in square brackets in equation (2.29) must be negative. Accordingly,

2.31 $$\cot^{2}\psi >\frac{2\varepsilon_{A}^{s}\left(\varepsilon_{A}^{s}+\varepsilon_{B}\right)}{\left(\varepsilon_{B}-\varepsilon_{A}^{s}\right)\left(\varepsilon_{A}^{s}+\varepsilon_{A}^{t}\right)}.$$

Also, the argument of the square root in equation (2.29) must be positive. Accordingly,

2.32 $$\cot^{2}\psi <\frac{\varepsilon_{A}^{s}}{\varepsilon_{B}-\varepsilon_{A}^{s}}.$$

By combining inequalities (2.31) and (2.32), we obtain

2.33 $$\frac{2\varepsilon_{A}^{s}\left(\varepsilon_{A}^{s}+\varepsilon_{B}\right)}{\left(\varepsilon_{B}-\varepsilon_{A}^{s}\right)\left(\varepsilon_{A}^{s}+\varepsilon_{A}^{t}\right)}<\frac{\varepsilon_{A}^{s}}{\varepsilon_{B}\;-\varepsilon_{A}^{s}},$$

which reduces to $2\varepsilon_{B}<\varepsilon_{A}^{t}-\varepsilon_{A}^{s}$. Also, for DV surface waves we have

2.34 $$\alpha_{B}=-ik_{0}\sqrt{\varepsilon_{A}^{s}\sec^{2}\psi -\varepsilon_{B}}.$$

Therefore, a DV surface wave exists only when the inequality $\varepsilon_{A}^{s}>\varepsilon_{B}\cos^{2}\psi$ is satisfied.

To summarize, it is essential for DV surface-wave propagation that the three inequalities

**Figure 2.**
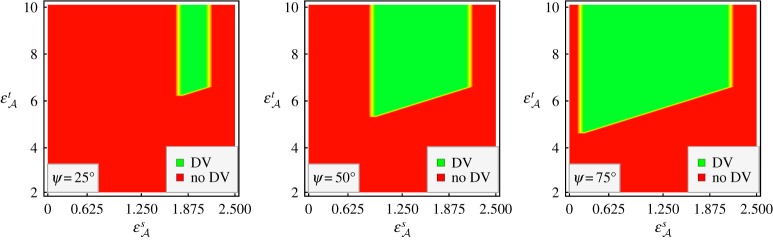
Portions of the $\varepsilon_{A}^{s}-\varepsilon_{A}^{t}$ plane that support DV surface-wave propagation and that do not support DV surface-wave propagation, for *ψ*∈{25°, 50°, 75°} with $\varepsilon_{B}=2.15$. (Online version in colour.)

2.35 $$\begin{array}{rl} & \varepsilon_{A}^{s}>\varepsilon_{B}\cos^{2}\psi \\ & \varepsilon_{A}^{t}-\varepsilon_{A}^{s}>2\varepsilon_{B}\\ \;\mathrm{and}\; & \varepsilon_{B}>\varepsilon_{A}^{s} \end{array}\}$$

are satisfied. These three inequalities impose quite severe constraints on the two partnering mediums to jointly support DV surface-wave propagation. The portion of the $\varepsilon_{A}^{s}-\varepsilon_{A}^{t}$ plane in which DV surface-wave propagation is allowed for *ψ*∈{25°, 50°, 75°} and $\varepsilon_{B}=2.15$ is identified in figure 2. It is clear that a strongly uniaxial medium A is needed for DV surface-wave propagation. In addition, the range of $\varepsilon_{A}^{s}$ that supports DV surface-wave propagation

— decreases continuously as *ψ* decreases, vanishing in the limit *ψ* → 0, and
— extends to $\left(0,\varepsilon_{B}\right)$ in the limit *ψ* → *π*/2.

For comparison, analysis of the dispersion equation (2.27) for the non-singular case reveals that the inequalities [2,4]

2.36 $$\varepsilon_{A}^{t}>\varepsilon_{B}>\varepsilon_{A}^{s}$$

must be satisfied for Dyakonov surface-wave propagation.

### (e) Analytical solutions of the Dyakonov–Voigt dispersion equation

The dispersion equation (2.29) can be solved analytically for one of four variables as follows.

— If $\varepsilon_{A}^{t}$, $\varepsilon_{B}$, and *ψ*∈(0, *π*/2) are fixed, then DV surface-wave propagation is possible only if
  2.37 $$\varepsilon_{A}^{s}=\frac{\sec^{2}\psi }{12}\left[t_{1}+\frac{2t_{2}}{{\left(2t_{3}+48\sqrt{6t_{4}t_{5}}\right)}^{\left(1/3\right)}}+{\left(2t_{3}+48\sqrt{6t_{4}t_{5}}\right)}^{\left(1/3\right)}\right],$$
  wherein the parameters
  2.38 $$\begin{array}{rl} t_{1} & =10\varepsilon_{B}-12\varepsilon_{A}^{t}+\left(4\varepsilon_{A}^{t}-6\varepsilon_{B}\right)\cos2\psi , \end{array}$$
  2.39 $$\begin{array}{rl} t_{2} & =71{\left(\varepsilon_{B}\right)}^{2}-126\varepsilon_{B}\varepsilon_{A}^{t}+67{\left(\varepsilon_{A}^{t}\right)}^{2}-4\left[15{\left(\varepsilon_{B}\right)}^{2}-34\varepsilon_{B}\varepsilon_{A}^{t}+15{\left(\varepsilon_{A}^{t}\right)}^{2}\right]\cos2\psi \\ & \;+\left[-3{\left(\varepsilon_{B}\right)}^{2}+6\varepsilon_{B}\varepsilon_{A}^{t}+{\left(\varepsilon_{A}^{t}\right)}^{2}\right]\cos4\psi , \end{array}$$
  2.40 $$\begin{array}{rl} t_{3} & =2\left[475{\left(\varepsilon_{B}\right)}^{3}-1359{\left(\varepsilon_{B}\right)}^{2}\varepsilon_{A}^{t}+1365\varepsilon_{B}{\left(\varepsilon_{A}^{t}\right)}^{2}-441{\left(\varepsilon_{A}^{t}\right)}^{3}\right]\\ & \;-3\left[345{\left(\varepsilon_{B}\right)}^{3}-1061{\left(\varepsilon_{B}\right)}^{2}\varepsilon_{A}^{t}+1023\varepsilon_{B}{\left(\varepsilon_{A}^{t}\right)}^{2}-347{\left(\varepsilon_{A}^{t}\right)}^{3}\right]\cos2\psi \\ & \;+6\left[15{\left(\varepsilon_{B}\right)}^{3}-51{\left(\varepsilon_{B}\right)}^{2}\varepsilon_{A}^{t}+65\varepsilon_{B}{\left(\varepsilon_{A}^{t}\right)}^{2}-21{\left(\varepsilon_{A}^{t}\right)}^{3}\right]\cos4\psi \\ & \;+\left[27{\left(\varepsilon_{B}\right)}^{3}-63{\left(\varepsilon_{B}\right)}^{2}\varepsilon_{A}^{t}+45\varepsilon_{B}{\left(\varepsilon_{A}^{t}\right)}^{2}-{\left(\varepsilon_{A}^{t}\right)}^{3}\right]\cos6\psi , \end{array}$$
  2.41 $$\begin{array}{rl} t_{4} & =2\left[105{\left(\varepsilon_{B}\right)}^{4}-151{\left(\varepsilon_{B}\right)}^{3}\varepsilon_{A}^{t}+17{\left(\varepsilon_{B}\varepsilon_{A}^{t}\right)}^{2}+67\varepsilon_{B}{\left(\varepsilon_{A}^{t}\right)}^{3}+6{\left(\varepsilon_{A}^{t}\right)}^{4}\right]\\ & \;+\left[-263{\left(\varepsilon_{B}\right)}^{4}+547{\left(\varepsilon_{B}\right)}^{3}\varepsilon_{A}^{t}-225{\left(\varepsilon_{B}\varepsilon_{A}^{t}\right)}^{2}+49\varepsilon_{B}{\left(\varepsilon_{A}^{t}\right)}^{3}+16{\left(\varepsilon_{A}^{t}\right)}^{4}\right]\cos2\psi \\ & \;+2\left[23{\left(\varepsilon_{B}\right)}^{4}-97{\left(\varepsilon_{B}\right)}^{3}\varepsilon_{A}^{t}+135{\left(\varepsilon_{B}\varepsilon_{A}^{t}\right)}^{2}-43\varepsilon_{B}{\left(\varepsilon_{A}^{t}\right)}^{3}+2{\left(\varepsilon_{A}^{t}\right)}^{4}\right]\cos4\psi \\ & \;+\left[7{\left(\varepsilon_{B}\right)}^{4}-19{\left(\varepsilon_{B}\right)}^{3}\varepsilon_{A}^{t}+17{\left(\varepsilon_{B}\varepsilon_{A}^{t}\right)}^{2}-\varepsilon_{B}{\left(\varepsilon_{A}^{t}\right)}^{3}\right]\cos6\psi \end{array}$$
  2.42 $$\begin{array}{r} \;\mathrm{and}\;t_{5}=-{\left(\varepsilon_{B}-\varepsilon_{A}^{t}\right)}^{2}\cos^{4}\psi \sin^{2}\psi ; \end{array}$$
— If $\varepsilon_{A}^{s}$, $\varepsilon_{B}$ and *ψ*∈(0, *π*/2) are fixed, then DV surface-wave propagation is possible only if
  2.43 $$\varepsilon_{A}^{t}=\frac{\varepsilon_{A}^{s}\left(\varepsilon_{A}^{s}-\varepsilon_{B}\right)+2\left(\varepsilon_{A}^{s}+\varepsilon_{B}\right)\sqrt{\varepsilon_{A}^{s}}\;\tan\psi \left(\sqrt{\varepsilon_{A}^{s}}\tan\psi +\sqrt{\varepsilon_{A}^{s}\sec^{2}\psi -\varepsilon_{B}}\right)}{\varepsilon_{B}-\varepsilon_{A}^{s}}.$$
— If $\varepsilon_{A}^{s}$, $\varepsilon_{A}^{t}$ and *ψ*∈(0, *π*/2) are fixed, then DV surface-wave propagation is possible only if
  2.44 $$\varepsilon_{B}^{}=\frac{1}{32\varepsilon_{A}^{s}}\left\{4t_{6}-\left(\varepsilon_{A}^{s}+\varepsilon_{A}^{t}\right)\csc^{2}\psi \left[4\left(\varepsilon_{A}^{s}+\varepsilon_{A}^{t}\right)-\sqrt{2\left(t_{7}+t_{8}\right)}\right]\right\},$$
  wherein the parameters
  2.45 $$\begin{array}{rl} & t_{6}={\left(\varepsilon_{A}^{t}\right)}^{2}+6\varepsilon_{A}^{s}\varepsilon_{A}^{t}-3{\left(\varepsilon_{A}^{s}\right)}^{2}, \end{array}$$
  2.46 $$\begin{array}{rl} & t_{7}=\cos4\psi \left[{\left(\varepsilon_{A}^{t}\right)}^{2}+10\varepsilon_{A}^{s}\varepsilon_{A}^{t}-7{\left(\varepsilon_{A}^{s}\right)}^{2}\right] \end{array}$$
  2.47 $$\begin{array}{rl} \;\mathrm{and}\; & t_{8}=4\cos2\psi \left(\varepsilon_{A}^{t}-3\varepsilon_{A}^{s}\right)\left(5\varepsilon_{A}^{s}+\varepsilon_{A}^{t}\right)+75{\left(\varepsilon_{A}^{s}\right)}^{2}-2\varepsilon_{A}^{s}\varepsilon_{A}^{t}+3{\left(\varepsilon_{A}^{t}\right)}^{2}. \end{array}$$
— If $\varepsilon_{A}^{s}$, $\varepsilon_{A}^{t}$ and $\varepsilon_{B}$ are fixed, then DV surface-wave propagation is possible only if
  2.48 $$\psi =\mathrm{arccot}\left[\frac{2}{\varepsilon_{A}^{s}+\varepsilon_{A}^{t}}\sqrt{\frac{\varepsilon_{A}^{s}\left(\varepsilon_{B}-\varepsilon_{A}^{t}\right)\left(\varepsilon_{B}+\varepsilon_{A}^{s}\right)}{\varepsilon_{A}^{s}-\varepsilon_{B}}}\right].$$

The surface wavenumber *q* for a DV surface wave is given by equation (2.14).

## 3. Illustrative numerical studies on Dyakonov–Voigt surface-wave propagation

We now examine the analytical solutions of the dispersion equation (2.29) for DV surface waves by means of representative numerical examples. For these computations, it is necessary to choose relative-permittivity parameters for mediums A and B such that the three inequalities (2.35) are satisfied. Owing to the symmetries of the dispersion equation (2.29), results need be presented for only 0 < *ψ* < *π*/2.

Let us begin with the the phase speed

**Figure 3.**
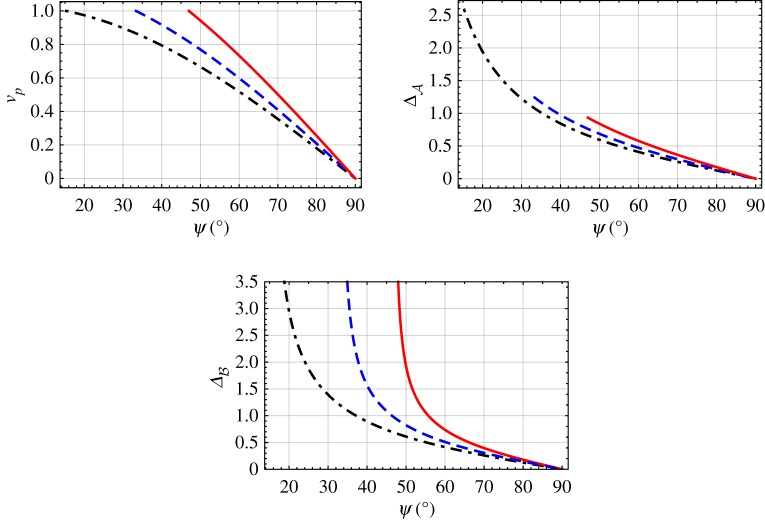
Relative phase speed *v*_*p*_ and normalized penetration depths $\Delta_{A}$ and $\Delta_{B}$, plotted versus *ψ*∈(0, *π*/2) for $\varepsilon_{A}^{s}=1$ (solid curves), 1.5 (dashed curves) and 2 (dash-dotted curves), with $\varepsilon_{B}=2.15$. (Online version in colour.)

3.1 $$v_{p}=\frac{k_{0}\sqrt{\varepsilon_{B}}}{q},$$

relative to the phase speed in unbounded medium B. In figure 3, *v*_*p*_ is plotted versus *ψ*∈(0, *π*/2) for $\varepsilon_{A}^{s}\in \{1,1.5,2\}$ with $\varepsilon_{B}=2.15$, using *q* determined from equation (2.14). The DV surface wave exists for *ψ*∈(47.00°, 90°) when $\varepsilon_{A}^{s}=1$, for *ψ*∈(33.36°, 90°) when $\varepsilon_{A}^{s}=1.5$, and for *ψ*∈(15.32°, 90°) when $\varepsilon_{A}^{s}=2$. The relative phase speed steadily approaches zero as the angle *ψ* approaches *π*/2. Furthermore, in the limit as *ψ* approaches its smallest value, we have *v*_*p*_ → 1.

Also provided in figure 3 are corresponding plots of the normalized penetration depths in mediums A and B, namely [2]

3.2 $$\begin{array}{rl} \Delta_{A} & =\frac{k_{0}}{\text{Im}\left\{\alpha_{A}\right\}}\\ \;\mathrm{and}\;\Delta_{B} & =\frac{k_{0}}{-\text{Im}\left\{\alpha_{B}\right\},} \end{array}\}$$

respectively, with $\alpha_{A}$ determined using equation (2.15) and $\alpha_{B}$ determined using equation (2.22). Both penetration depths steadily approach zero as the angle *ψ* approaches *π*/2. In the limit *ψ* → *π*/2, we have $\Delta_{A}=\Delta_{B}=0$ and also *v*_*p*_ = 0. Furthermore, as *ψ* approaches its smallest value, $\Delta_{B}$ becomes unbounded, whereas $\Delta_{A}$ increases but remains bounded.

**Figure 4.**
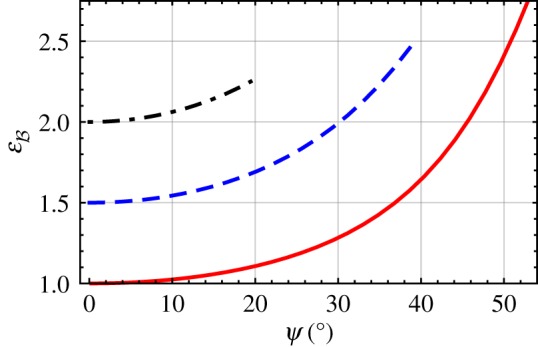
Relative permittivity $\varepsilon_{B}$ plotted versus *ψ*∈(0, *π*/2) for $\varepsilon_{A}^{s}=1$ (solid curve), 1.5 (dashed curve) and 2 (dash-dotted curve), with $\varepsilon_{A}^{t}=6.5$. (Online version in colour.)

In figure 4, the solution for $\varepsilon_{B}$ given in equation (2.44) is plotted versus *ψ* for 0 < *ψ* < *π*/2, with $\varepsilon_{A}^{s}\in \{1,1.5,2\}$ and $\varepsilon_{A}^{t}=6.5$. When $\varepsilon_{A}^{s}=1$, the DV surface wave exists for *ψ*∈(0°, 52.91°), and $\varepsilon_{B}$ uniformly increases from 1 to 2.75 as *ψ* increases. When $\varepsilon_{A}^{s}=1.5$, the DV surface-wave propagation is possible for *ψ*∈(0°, 39.23°); furthermore, as *ψ* increases, $\varepsilon_{B}$ uniformly increases from 1.5 to 2.5. When $\varepsilon_{A}^{s}=2$, the DV surface wave propagates for *ψ*∈(0°, 19.47°), and as *ψ* increases the value of $\varepsilon_{B}$ uniformly increases from 2 to 2.25.

**Figure 5.**
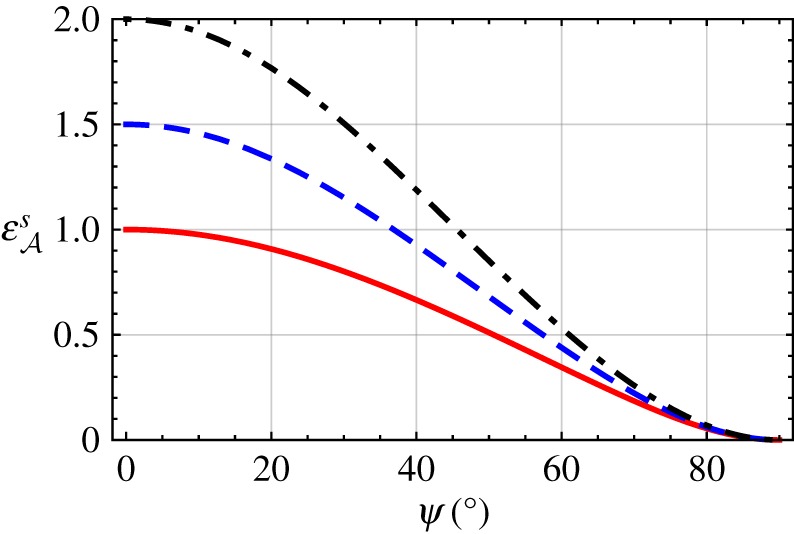
Relative permittivity $\varepsilon_{A}^{s}$ plotted versus *ψ*∈(0, *π*/2), for $\varepsilon_{A}^{t}=6.5$ with $\varepsilon_{B}=1$ (solid curve), 1.5 (dashed curve) and 2 (dash-dotted curve). (Online version in colour.)

The solution for $\varepsilon_{A}^{s}$ provided in equation (2.37) is plotted in figure 5 versus *ψ*∈(0, *π*/2) for $\varepsilon_{A}^{t}=6.5$ with $\varepsilon_{B}\in \{1,1.5,2\}$. The DV surface-wave propagation is possible for all values of *ψ*∈(0, *π*/2). Furthermore, $\varepsilon_{A}^{s}$ decreases uniformly as *ψ* increases, taking the value of *ε*_*B*_ in the limit *ψ* → 0, and becoming null valued in the limit *ψ* → *π*/2, for all values of $\varepsilon_{B}$.

**Figure 6.**
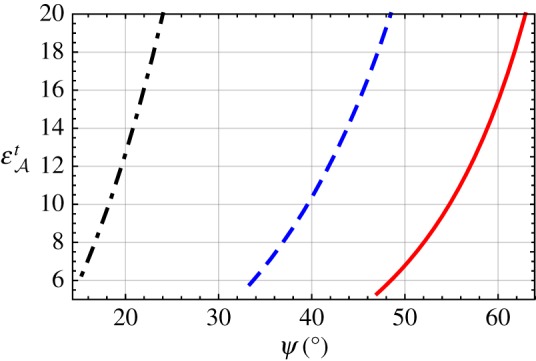
Relative permittivity $\varepsilon_{A}^{t}$ plotted versus *ψ*∈(0, *π*/2), with $\varepsilon_{B}=2.15$ for $\varepsilon_{A}^{s}=1$ (solid curve), 1.5 (dashed curve) and 2 (dash-dotted curve). (Online version in colour.)

Next we turn to the solution for $\varepsilon_{A}^{t}$ provided by equation (2.43). In figure 6, $\varepsilon_{A}^{t}$ is plotted versus *ψ*∈(0, *π*/2) with $\varepsilon_{A}^{s}\in \{1,1.5,2\}$ and $\varepsilon_{B}=2.15$. When $\varepsilon_{A}^{s}=1$, DV surface waves exist for *ψ*∈(47.00°, 90°); as *ψ* increases, the value of $\varepsilon_{A}^{t}$ uniformly increases from 5.3 and becomes unbounded as *ψ* approaches *π*/2. When $\varepsilon_{A}^{s}=1.5$, the solution exists for *ψ*∈(33.36°, 90°) and, as *ψ* increases, the value of $\varepsilon_{A}^{t}$ uniformly increases from 5.8 and becomes unbounded as *ψ* approaches *π*/2. When $\varepsilon_{A}^{s}=2$, DV surface-wave propagation is possible for *ψ*∈(15.32°, 90°); the value of $\varepsilon_{A}^{t}$ uniformly increases from 6.3 as *ψ* increases, becoming unbounded as *ψ* approaches *π*/2.

**Figure 7.**
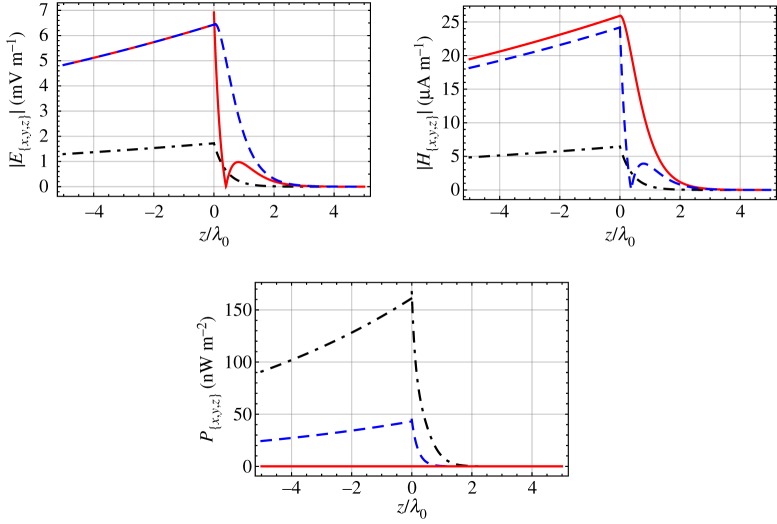
Magnitudes of the Cartesian components of $\underline{E}(z{\hat{\underline{u}}}_{z})$ and $\underline{H}(z{\hat{\underline{u}}}_{z})$, and the Cartesian components of $\underline{P}(z{\hat{\underline{u}}}_{z})$, plotted versus *z*/*λ*_0_, for $\varepsilon_{A}^{s}=2$, $\varepsilon_{A}^{t}=6.5$, $\varepsilon_{B}=2.15$ and *ψ* = 15.32°, with $C_{B1}=1\;{V\;m}^{-1}$. Key: *x*-directed components, dash-dotted curves; *y*-directed components, dashed curves; *z*-directed components, solid curves. (Online version in colour.)

In order to shed further light on the features of DV surface waves, spatial profiles of the magnitudes of the Cartesian components of the electric and magnetic phasors are provided in figure 7 for $\varepsilon_{A}^{s}=2$, $\varepsilon_{A}^{t}=6.5$ and $\varepsilon_{B}=2.15$. From equation (2.48), the corresponding propagation angle is *ψ* = 15.32°. The amplitude $C_{B1}=1\;{V\;m}^{-1}$ is fixed. The magnitudes of the components of the electric and magnetic field phasors show an apparent exponential decay as the distance |*z*| from the interface increases. The rate of decay is considerably faster in medium A than in medium B. Thus, we infer that the linear term in equation (2.19) is dominated by the exponentially decaying terms. The localization of the DV surface waves can also be appreciated from the spatial profiles provided in figure 7 of the Cartesian components of the time-averaged Poynting vector

3.3 $$\underline{P}(\underline{r})=\frac{1}{2}\text{Re}\left[\underline{E}(\underline{r})\times {\underline{H}}^{*}(\underline{r})\right],$$

where the asterisk denotes the complex conjugate. We observe that there is no energy flow in directions normal to the interface *z* = 0; that is, energy flow is restricted to directions parallel to the interface plane.

## 4. Closing remarks

A new type of electromagnetic surface wave—called a Dyakonov–Voigt (DV) surface wave—has been found theoretically. The propagation of these surface waves is guided by the planar interface of an isotropic dielectric medium and a uniaxial dielectric medium whose optic axis lies in the interface plane, provided that certain constraints on the constitutive parameters of the partnering mediums, specified by inequalities (2.35), are satisfied.

The DV surface wave has some features in common with the Dyakonov surface wave [3,4]. Most notably, surface waves of both types are guided by the planar interface of non-dissipative dielectric mediums, one of which is anisotropic. But there are fundamental differences between them too. Most notably, the decay in the amplitude of DV surface waves with distance from the interface in the anisotropic partnering medium involves a linear term per equation (2.19) (as well as exponential terms), whereas the corresponding decay for Dyakonov surface waves only involves exponential terms per equation (2.13). Furthermore, the constitutive-parameter regime that supports DV surface waves, as characterized by inequalities (2.35), is not the same as the constitutive parameter regime that supports Dyakonov surface waves, as characterized by inequalities (2.36). And, whereas Dyakonov surface waves propagate for a range of directions in each quadrant of the interface plane [8,9], DV surface waves propagate in only one direction in each quadrant of the interface plane.

Voigt-wave propagation is possible in an unbounded anisotropic dielectric medium only if the relative permittivity dyadic of that medium is non-Hermitian [31]. The non-Hermitian nature of the relative permittivity dyadic may reflect the dissipative [15] or active [30] nature of the medium. On the other hand, DV surface-wave propagation can be supported by partnering dielectric mediums whose relative permittivity dyadics are Hermitian but the matrixes $\left[{\underline{\underline{P}}}_{A}\right]$ and $\left[{\underline{\underline{P}}}_{B}\right]$ are both non-Hermitian, as reported in the preceding sections.

Lastly, while the canonical boundary-value problem investigated herein is a useful idealization, it does not directly shed light upon the excitation of DV surface waves in a practical configuration, such as the prism-coupled configuration [2]. This is a matter for future study, which will deliver insights into the polarization states of DV surface waves.

## Data accessibility

This article has no additional data.
